# Intentional Non-Adherence in Schizophrenia: The Influence of Antipsychotics’ Sedative Side-Effects A First-Person Account

**DOI:** 10.1093/schizbullopen/sgaf020

**Published:** 2025-10-04

**Authors:** Jensen Gert

**Affiliations:** Independent

*Sedation is a common occurrence with conventional antipsychotics, but its effects can sometimes be mistaken for the negative symptoms of schizophrenia, such as avolition, amotivation, asociality, withdrawal, and anhedonia.* ^1^

.

## Introduction

The question of why some schizophrenia patients resist antipsychotic medicine, an issue referred to as non-adherence or non-compliance, has been raised several times.^2–5^ From my own experiences, sedative side-effects may be an important but not fully understood reason for such non-adherence. To an outside observer, sedative side-effects may seem akin to negative symptoms, but from a first-person perspective, they have very different experiential qualities and consequences to mental functioning.

In this paper, I argue that sedative side-effects can cause a loss of emotional cognition, and that this loss can lead to an intentional rejection of antipsychotic medicine.

### Autobiographic Note

I am a male in my late thirties diagnosed with ICD-10 schizophrenia, seemingly following a relapsing course. I am especially struggling with negative symptoms. Disorganized, positive, and cognitive symptoms also create regular challenges. I am a previous user of psychiatric services and an episodic user of antipsychotics.

### Defining Sedative Side-effects, Emotional Cognition, and Negative Symptoms

I use the term emotional cognition to refer to the ability of navigating one’s inner lifeworld through “subjective feeling” (i.e. an ability of feeling such that one can intuit meaning of phenomena and how these are linked).

Sedative side-effect refers to the subjective experience of sedation, drowsiness, and a specific loss of mental function. Hence, “the slowing, or closing down, of emotion and cognition, namely ‘Drowsiness, feeling tired, sedation’ ‘Loss of motivation’, ‘Slowed thoughts’, ‘Emotional numbing’ and ‘Difficulty concentrating’” (^4^ p. 179).

Negative symptoms refer to anhedonia, flat affect, asociality, avolition, and alogia.^6^ More specifically, I am referring to the subjective experience of anhedonia, that is, the lack of ability to feel or recall pleasure, and of flat affect, that is, the decreased verbal or non-verbal expression of emotions (which is usually considered an expressive feature).

### Sedative Side-effects and Intentional Non-adherence

Sedative side-effects can have a distinct effect on emotional cognition that is different in experiential quality than that of negative symptoms. This experience of sedative side-effects on emotional cognition can cause non-adherence.

It is important to distinguish between the experience of negative symptoms and of sedative side-effects in terms of how these are experienced as a loss of function. While negative symptoms can have the effect of diminishing one’s motivation, will, and energy to act, sedative side-effects of antipsychotics can have the effect of completely removing certain modes of experiencing. For example, negative symptoms can bring about a feeling of emptiness, where sedative side-effects can remove the ability to feel altogether, so that there is no feeling at all. In the same vein, negative symptoms can manifest as discomfort in social situations, but sedation can “put to sleep” one’s ability to feel “something” in or about a social situation.

The central issue with such a removal of emotional cognition through sedative side-effects is that there is a lack of access to certain aspects of one’s emotional ability to “navigate” one’s mental landscape. This can be a difficult sensation to explain to outside observers, as this seems very alike the disturbance that may follow from anhedonia. However, where anhedonia can create discomfort, sadness, dysthymia (sub-depression-like state), such a negative emotional state is a type of emotional cognition in the sense that one can still feel and navigate facets of one’s inner lifeworld. Importantly, it does not turn off the ability to have a sense of meaning in one’s inner lifeworld, for example, at least there is a sense of irritation, frustration, a feeling of “lack”, etc. There is “something” to which one can relate, “something” one can reflect upon and struggle with. Sedative side-effects are different. Where negative symptoms can leave a sense of emptiness, sedative side-effect can remove the sense altogether such that there is nothing left to sense. This can create challenges in contexts such as psychotherapy, metacognition, and “self-disturbance”.

#### Psychotherapy

Sedative side-effects can make psychotherapy difficult when emotional feedback is reduced or eliminated. The ability to use emotion to intuit the meaning or importance of one’s own thoughts can be argued to be of vital importance in many types of psychotherapy. For example, in the context of cognitive behavioral therapy, sedative side-effects could cause difficulties in navigating so-called hot cognitions, (^6^ p. 71), that is, cognition signified as important to the patient because of its emotional feedback.

#### Metacognition

Sedative side-effects may cause a disturbance to metacognition, that is, social cognition. From my own experiences of using one type of second-generation antipsychotic over a period of five months, there was a gradual loss of empathy. This can be difficult to interpret from an outside perspective, as this effect can look like difficulties in metacognition common among some schizophrenia patients.^7^ From an outside perspective, positive and negative symptoms may resemble sedative side-effects in how they can disrupt one’s ability to form sympathetic relations. However, the experiential process is very different from an internal perspective. Many of the difficulties in metacognition as a result from negative and positive symptoms are, in my experience, founded on an inability to establish an “If I was in his place, then I would feel X” because the state of being an “I” is fundamentally changed. It is changed either in the sense that the experience of being a “self” is gone, or in the sense that the inner lifeworld is so changed by positive symptoms that it has become alien to the shared social reality, and it is therefore impossible to mirror oneself in the place of “the other”. There is no “I” who can “feel like him”. However, the experiential quality sedative side-effects is different: whereas negative and positive symptoms can be said to disrupt metacognition, sedative side-effects can be experienced as eliminating the ability to form emotionally guided metacognition.

#### Self-disturbance

Schizophrenia can be characterized as a self-disorder.^8–12^ However, there is, in my experience, a significant qualitative difference between the disturbance of the self in schizophrenia versus disturbance of self caused by sedative side-effects. As a person with schizophrenia, my experience of self is detached from an inner lifeworld in the sense that most of the experiences I have internally, such as thoughts, ideas, emotions, and intentions, are not felt as “mine”. They are rather experienced as phenomena in themselves in the sense that a thought manifests in experience just as a chair I encounter in the outer lifeworld. To say that this chair I perceive has a sense of “mineness” seems just as absurd as to say that a thought I encounter in my inner lifeworld is “mine”. Thus, I often experience confusion when navigating my inner lifeworld, as much of it does not seem a natural part of what is “me” but rather a random occurrence. To be clear, there is a sense of me, but this sense of me is detached, observing from the outside, and often more at home in a particular “elsewhere”, often referred to as a “private solipsistic” world.^8^^,^^9^^,^^13–17^ The sedative side-effects can affect the inner lifeworld, potentially creating a different kind of disturbance to the self in the sense that elements that help navigate this inner lifeworld in relation to the self, (i.e. the ability to use emotion to intuit meaning or navigate one’s psyche) are reduced or lost. This can lead to a fragmentation of the inner lifeworld in the sense that the emotional cognition that helped “tie” the inner lifeworld together, such that the self can navigate it and make sense of it, can no longer do so. As a result, the inner lifeworld seems to split into disconnected parts, if these parts appear to the self at all. The sedative side-effects can, in this sense, cause a “blindness” to elements of the inner lifeworld.

## Discussion

Side-effects of medicine can create challenges that can be experienced as just as challenging as some psychotic symptoms, if not sometimes even more. Therefore, it can seem like a better alternative to reject medicine than to risk additional complications.

I have had many conversations on this topic with other veteran patients sharing my diagnosis, and I have found the challenges mentioned in this paper to be common. I do acknowledge that my account is far from complete and that experiences of sedative side-effects of medicine deserve further research. The effects of psychosis and medicine on one’s decision process, existential challenges, lifeworld, and non-adherence are far more complicated and nuanced than pointed to in this paper.
